# Women’s experiences and views of outpatient and inpatient induction of labor with oral misoprostol: A secondary qualitative study

**DOI:** 10.18332/ejm/172651

**Published:** 2023-11-17

**Authors:** Hanne A. Hægeland, Marianne G. Moi, Fride E. Austad, Hanna Oommen, Janne Rossen, Mirjam Lukasse

**Affiliations:** 1 Department of Nursing and Health Sciences, Faculty of Health and Social Sciences, University of South-Eastern Norway, Campus Vestfold, Norway; 2 Department of Obstetrics and Gynecology, Sørlandet Hospital HF, Kristiansand, Norway

**Keywords:** induction of labor, choice, shared decision-making, misoprostol, outpatient induction, women’s birth experiences

## Abstract

**INTRODUCTION:**

As labor induction rates continue to increase, so has the interest in performing induction in an outpatient setting for pregnancies defined as low-risk. Twenty women participated in the pilot study of a Randomized Controlled Trial (RCT) comparing inpatient and outpatient labor induction with oral misoprostol. This study aimed to explore women’s experiences of outpatient induction of labor and their views on this as an alternative method to inpatient labor induction.

**METHODS:**

Semi-structured interviews were conducted, from November 2021 to January 2022 with eight women randomized to outpatient induction and four women randomized to inpatient induction. Verbatim transcribed interviews were analyzed using Graneheim and Lundman’s content analysis.

**RESULTS:**

Three main categories emerged: the required framework around outpatient labor induction, what felt better at home and what felt safer at the hospital. To feel secure at home, women needed sufficient information, close follow-up while at home, and an easy-to-administer induction method. Outpatient labor induction gave women the opportunity of constant support from the partner and increased freedom of movement and self-expression. Some expressed relief over being randomized to inpatient labor induction, because of easy access to health providers, fetal monitoring, and not risking giving birth before arrival to the hospital. Women stressed the importance of being given a choice.

**CONCLUSIONS:**

Outpatient labor induction contributed to a positive birth experience and should be considered as an alternative for women with low-risk pregnancies. Shared decision-making, including the opportunity for women to change their mind, is essential as induction and early labor affects women’s whole childbirth experience.

## INTRODUCTION

Induction of labor is an obstetric intervention to start labor when the benefits of ending the pregnancy for mother and child are considered greater than the risks of continuing the pregnancy^1^. The most common reasons for induction after 37 weeks of gestation are post maturity and spontaneous rupture of membranes without contractions^2^. The number of pregnant women experiencing labor induction is rising internationally^3^. In Norway, the rate has increased from 11% in 2000 to 28.3% in 2021^4^. Factors influencing this increase include expanded indications and a shift in the gestational age when labor induction is recommended for post maturity^2^. In addition, evidence suggests that induction of labor may not increase the risk of negative outcomes such as operative delivery and admission to intensive care for the newborn^2^.

Several different methods exist for induction of labor. When the cervix is unripe, it is common to start the process of induction with either a balloon catheter placed within the cervix or prostaglandins^1^. Prostaglandins (for example misoprostol) affect the cervix by making it softer and shorter, in addition to stimulate uterine contractions^1^. A Cochrane review found that oral misoprostol and vaginal misoprostol are both effective at achieving vaginal birth, but oral misoprostol resulted in less cesarean sections^5^. Moreover, oral use is particularly attractive because of an easy and non-invasive administration and strong evidence suggests oral administration as the first choice^5^. The World Health Organization (WHO) recommends 25 mcg misoprostol orally every two hours until contractions start^6^. Use of prostaglandins is the most common method for cervical ripening in Norway^4^.

Traditionally, induction of labor is performed at the hospital, allowing an observation of both the mother and fetus. However, several high-income countries are now offering outpatient labor induction as an alternative to pregnant women with low-risk pregnancies^7-11^. Staying at home in anticipation of the onset of childbirth may improve a woman’s experience of labor induction and birth^12^. Being in a familiar setting with a partner or family can provide a feeling of support and calmness. At home, women are likely to experience greater freedom to move freely, to eat and drink what they want and when they want, to sleep in familiar surroundings, express and be themselves as well as continuing with daily routines^2,10,11^.

Until recently, the national guidelines for induction of labor in Norway did not include the option of outpatient induction with oral misoprostol^1^. However, the increasing interest among birth attendants regarding outpatient labor induction with oral misoprostol for low-risk pregnancies has led to a couple of studies to investigate the feasibility and safety of this practice, including an external Pilot Randomized Controlled Trial (RCT) of twenty women. We aimed to explore pilot-participating women’s experiences of outpatient induction of labor with oral misoprostol and their views on outpatient induction of labor as an alternative method to inpatient induction of labor.

## METHODS

### Participants

We conducted twelve semi-structured interviews from 29 November 2021 to 12 January 2022; eight with women who had an outpatient labor induction and four with women who had an inpatient labor induction. Participants were recruited from the Pilot Randomized Controlled Trial on Oral Misoprostol for Outpatient Induction of Labor (ClinicalTrials.gov Identifier: NCT04596397). The external pilot study aimed to assess the feasibility of conducting a multicenter randomized controlled trial of outpatient versus inpatient induction of labor with oral misoprostol and was performed from 15 November 2020 to 15 March 2021. Twenty women consented to participate in the pilot study and to be contacted for an interview at a later stage. The inclusion criteria for the pilot study defined women as low-risk and included a single pregnancy, at term with a vertex presentation. Furthermore, women had to be aged 18–40 years, understand and read Norwegian, have no cognitive barriers, normal ultrasound including fetal movements, amniotic fluid, estimated fetal weight ±15% (≤10 and ≥90 percentile) and normal doppler peak systolic index in the umbilical cord artery and the cerebri media artery, normal cardiotocography, body mass index 18–35 kg/m^2^, no signs of infection or health problems, and distance to hospital about one hour. Exclusion criteria for the study were: premature rupture of membranes, previous cesarean section or operation on uterus, fetal anomaly or chromosome/genetic disorder, pregnancy complications such as preeclampsia and diabetes (insulin dependent) or other conditions where changes in fetal heart rate during labor is suspected.

### Pilot study induction procedure

All twenty women received the first 25 mcg misoprostol orally at the hospital. After two hours, the women in the outpatient group could go home if they did not have contractions and the cardiotocography (CTG) was normal. At home, these women were instructed to take 25 mcg misoprostol orally every two hours until the contractions started or they experienced increasing intensity of pain commonly observed with the progression of labor with a maximum of six tablets in 24 hours. If labor was not established after 48 hours (12 tablets), the women were admitted to hospital to continue the induction process. All women in the outpatient group received written information with instructions and a direct telephone number to one antenatal ward midwife in the hospital. The women randomized to the inpatient group also received 25 mcg misoprostol orally every two hours, maximum of six tablets in 24 hours, for 48 hours or until labor was established. In addition, they were monitored with CTG every four to six hours.

### Data collection

Data were collected by doing face-to-face interviews. Two semi-structured interview guides were created – one interview guide for the women in the outpatient group and another one for the women in the inpatient group.

The first six and the last three interviews were conducted by two persons (HAH and MGM). Interviews seven, eight and nine, were conducted by one person (HAH). The researchers benefited from the fact that two of them were involved in the interviews ensuring that all areas were covered. The interviews took place in the women’s homes. Notes were made during the interviews, to make the follow-up questions easier to remember, and to avoid interrupting the woman during the interview. Interviews lasted 30–45 min, were audio-recorded, and transcribed verbatim on the same or the following day. HAH and MGM transcribed jointly, dividing each interview in two parts. Data saturation was achieved after six interviews in the outpatient group, with the further two interviews confirming this. Four interviews provided sufficient material in the inpatient group. This was discussed and agreed upon by the first, second and last author.

### Method for the qualitative data analysis

Data analysis was conducted in collaboration by the first, second and last author to ensure the quality of the analysis^12^. We used the content analysis of Graneheim and Lundman^13^ which included the following steps. In step 1, a thorough review of the entire text to form an overall impression. In steps 2–4, meaning units were identified, condensed, and labelled with a code and were systematically put in a table. We marked the meaning units and the codes with different colors in the table to get a better overview and to see more easily what became sub-categories and categories. In steps 5 and 6, we divided codes into categories and sub-categories and results were written^14^. There were no conflicts in defining the categories and sub-categories which emerged from the data. The whole analysis process was an iterative process.

### Ethical approval

The study followed the Helsinki Protocol (WMA Declaration of Helsinki at www.wma.net). The study was approved by the Regional Committee for Ethics in Medical Research, Region South (104044) on 11.09.2020 and the Norwegian Data Inspectorate (552589) on 06.11.2020. All women invited to this study received a new information letter and informed consent was obtained before they participated in this secondary study. Women were ensured that their data were collected and stored in a way that protected their privacy and limited access to the researchers of the study only. Identifiable information, such as women’s name, age, and parity, was stored separately from the interviews, and results were anonymized.

## RESULTS

Our study included four primiparous and eight multiparous women and aged 24–39 years. All eight women in the outpatient induction group gave birth spontaneously with no adverse events such as sphincter rupture, postpartum hemorrhage >500 mL or Apgar score <7 after 5 min. One of the four women in the inpatient induction group had an acute cesarean section and postpartum hemorrhage >1000 mL. None of the four had adverse events such as sphincter rupture, or Apgar score <7 after 5 min.

The analysis resulted in three categories: the required framework around outpatient induction, what felt better at home, and what felt safer in hospital.

### The required framework around outpatient induction

Women commented on what they felt was necessary in order to be induced at home. This included sufficient information, close follow-up while being at home, an easy induction method, being given a choice, and the freedom to change their mind.

### Sufficient information

Women described the need for information about outpatient induction as early as possible, preferably already during the pregnancy consultation. Being given time to think and ask questions aided the decision-making process. Information had to be easy to understand and both verbal and written information was deemed necessary. Due to the COVID-19 pandemic women tended to attend antenatal care consultations on their own and became more or less alone in both receiving the information and making the decision of participating in the study. Women commented that the partner too should receive information from the midwife/doctor, so that the partner would receive the same information as they had. Women found it challenging to be responsible for passing on the information to their partner and reassuring them about outpatient labor induction. One woman suggested that the obstetrician called her partner so they that both received the same information:

*‘I was very confident with the information I received in advance.’* (Participant 2)

*‘Information about it – early on, I think. Maybe already at the community health center when you go to the midwife there, for instance at the 36-week check-up.’* (Participant 2)

The written information proved important once women were at home and the process of induction had started. Women appreciated detailed information about what should stop them from taking more misoprostol tablets as well as information about the open line to the hospital. Several women reported on how they followed the written instructions that they had received and that it was important to have the leaflet with written information at hand when they needed it:

*‘I think the information was clear and nice on the sheet. Really short and specific like.’* (Participant 3)

However, women commented that written information on its own was not always sufficient.

### Close follow-up while being at home

Midwives’ availability and having access to professional support over the phone 24/7 was essential for women to feel safe at home. Women were reassured by the midwives’ advice and guidance over the phone and by knowing that they were welcome at the hospital anytime. In addition, midwives’ competence and warm and respectful response was of great importance, in particular when unforeseen things happened:

*‘I got a midwife available that I could call at any time. They were very accessible, and they picked up the phone right away. Answered questions very reassuringly and I was constantly told that I could come [to the hospital] anytime.’* (Participant 4)

Women highlighted the importance of reaching the midwife directly when calling the hospital and their perception that the midwife knew about them. Furthermore, women felt it was advantageous that it was the same midwife whom they had communicated with by phone that met them when being admitted to the hospital. Several women expressed a desire to receive a phone call from a midwife to feel followed-up when being home:

*‘That the mothers are confident that ... that they’re being followed-up. That they are not forgotten when they go home … I think I would have appreciated that there were some phone calls, like … or that they would not just send me home and make me feel as if they had forgotten about me. That’s what it is all about – it is all about the confirmation that I’m safe. I think that’s important.’* (Participant 7)

### An easy induction method

Overall, women found the method used to induce labor with oral misoprostol easy. To take tablets was perceived as unproblematic. Tablets can be taken anywhere, as one woman pointed out, even while being at a restaurant. One woman appreciated the method being less invasive than other induction methods, gentle and not involving vaginal examinations. The method increased the feeling of selfcontrol:

*‘Everything was in control all the way. And I was in control, as I administered the tablets.’* (Participant 2)

*‘They [the tablets] had to be taken every two hours ... Other than that, there were no other instructions, as far as I know. So, that gives you a little freedom. “Okay, this is the only thing I have to do – to take those tablets and to be aware of how I’m doing”. Quite simple. Other than that, I can do whatever I want.’* (Participant 9)

Almost all women mentioned that they became uncertain about taking the next set of tablets once they started having contractions. Some women expressed that the guidelines were clear and detailed, while others wanted to contact the hospital for guidance. Women reported that a short conversation could be clarifying. Some women were afraid of bothering the health providers by calling them:

*‘I'm not a person who calls easily. So, it is always difficult to determine “has it gone too far - should I call now - or should I call now - or is it just nonsense”.’* (Participant 10)

### Being given a choice and the freedom to change one’s mind

Several women joined the study because it provided them with the opportunity to stay at home during the induction process, which was their preferred choice. However, some were relieved to be randomized for inpatient labor induction as this was what they really wanted. Women emphasized the importance of having a choice and that the decision was dependent on their own personal preference:

*‘It’s very important that the choice is 100% your own choice.’* (Participant 3)

*‘It is better with a choice rather than “this is how it is done”.’* (Participant 5)

Women appreciated the freedom to change their mind during the induction process:

*‘I was also told that, if I regret my decision … if I came home and did not think it was okay, then I could return to the hospital … I didn't have to be at home until the birth started.’* (Participant 1)

### What felt better at home

Women described what made their home a more comfortable place to be during the induction process. This included making the start of labor more natural and being guided by one’s own needs, and having constant support from the partner.

### Making the start of labor more natural and guided by one’s own needs

Women used a consistent series of positive ways to describe how they felt at home. They reported that they felt ‘more relaxed’ and ‘more calm’ in a ‘familiar setting’ and that there was a sense of ‘freedom of movement and self-expression’ compared to the hospital environment. Some women mentioned being more easily distracted from the induction process when at home and the feeling that time passed faster. Women kept busy with their usual routines at home, including watching television, cleaning the house, going to the store, doing homework with the children, while being in the early stages of labor. However, several women mentioned that having their children around was stressful and organizing childcare was crucial:

*‘All I see is benefits really … sleeping in your own bed with your own pillow and your own comforter and baby-hug-me ... your own bathroom … to shower in your own shower … and being able to go in the fridge when you’re hungry … yes, watching television … going for a walk or going to the stores. Making the time pass.’* (Participant 4)

*‘You have everything around you … my own things.’* (Participant 9)

In contrast to the home setting, the hospital environment was described as alien and unnatural. Women expressed that being in the hospital was associated with being bored, constantly meeting new midwives, being served tasteless food, lots of noise, being monitored, a lot of waiting, being lonely, and stuck in a room in a busy ward. Lack of privacy at the hospital and sharing a room with a stranger while being in early labor, were perceived as undesirable. Having the autonomy to decide what to eat, when to eat, when to sleep and what to do, provided women with a sense of control over the birth experience and was of great importance for women wanting to stay at home in the early stages of labor:

*‘It's not normal surroundings [at the hospital] at all. You are put out of action.’* (Participant 10)

*‘It won’t be personal if you have to share your (birth) experience with completely strangers.’* (Participant 2)

As in spontaneous start of labor, the women interpreted fetal movement as a sign of fetal wellbeing. Similar to spontaneous start of labor, women felt excitement at being at home while waiting for contractions to start and deciding to pack their bags for hospital admission. A couple of women mentioned that staying at home as long as possible was a bonus. Some women expressed that it was easier for the body to start labor when being relaxed at home:

*‘The advantage [of outpatient induction] is that it is much calmer and that the body gets the opportunity to contribute.’* (Participant 2)

### Having constant support from the partner

All of the women in the study expressed their desire to have their partner continuously present or available in early labor. Women reported that being with someone who knows you and your needs was of great importance and could not be replaced by a midwife. Women were aware that due to the COVID-19 restrictions, their partner was only allowed to visit at certain times at the hospital. Thus, being at home with the partner and going through the process together was particularly valued:

*‘[The partner] is a fundamental support. There’s something about being two – that you are not completely alone.’* (Participant 10)

*‘I let the guard down when he was around.’* (Participant 7)

In addition to emotional support, the partner provided the woman with practical support. It was easier for women to ask the partner about practical services than from a midwife. The partner was described as more comfortable with their role as supporter and less likely to feel redundant at home:

*‘When I am being induced at the hospital, I think he feels some kind of hopelessness - that there’s nothing he can do [to help]. Whereas at home, we can be ourselves. And when contractions start, he asks what he can do for me. I answer “nothing” ... Then he can accept it and not feel so stupid.’* (Participant 10)

One woman mentioned that her partner was stressed by the outpatient induction and that she had to comfort him.

### What felt safer at hospital

Women described being in the hospital as being associated with a greater sense of safety through having easy access to health providers, fetal monitoring, and no risk of giving birth before arrival to hospital.

### Easy access to health providers

Having the midwives physically nearby, even if the midwives were not constantly present, made women feel safer at the hospital. The hospital could provide the women with medical expertise in case of emergency. In addition, knowing that the health providers had the overall responsibility of the unborn baby and the induction process, was reassuring. The women appreciated having the chance to ask questions more directly and having the opportunity to get confirmation on the stage of labor:

*‘But it’s a bit like that, with the hospital – that you feel taken care of anyway, even if they do not have time, they are present. If you ring the big red bell, they will run to you, that's how it is.’* (Participant 10)

### Fetal monitoring

All women in our study with inpatient induction found fetal monitoring beneficial. Fetal monitoring was valuable in easing their fears about the baby’s safety and giving the women the confirmation they needed in order to feel safe. Although everyone in the outpatient group agreed about fetal monitoring being a bonus, some women mentioned that CTG was not something they missed while being at home. Several women expressed that this intervention could sometimes be disturbing in terms of limiting their freedom of movement, that it could be time consuming and unnecessary when everything had been normal so far. However, one woman mentioned that she was extra aware of fetal movement at home:

*‘It was [positive] to get monitoring and constantly being able to ask about something if I was wondering about something. So, for me, it's really the confirmation that everything was fine, that was positive in itself.’* (Participant 6)

### No risk of giving birth before arrival

Finding the ‘correct time’ for admission was important. The women expressed having concerns about the distance to the hospital, measured both in kilometers and minutes. The fear of giving birth before arriving at the hospital was a concern for half of the participating women in the study, both of those randomized to outpatient and inpatient labor induction. Being at the hospital relieved them of this responsibility:

*‘[Concerned about] not arriving in time. To give birth at home, or in the car, or you arrive at [the hospital] and then you have... come so far that the baby is almost born. There is something about having a controlled birth.’* (Participant 5)

## DISCUSSION

Women in our study were positive about outpatient labor induction as long as they received sufficient information, were followed-up while being at home, in addition to an easy induction method. It was important being given a choice and having the opportunity to change their mind during the induction process. Being at home gave women the opportunity of constant support from the partner, the increased freedom of movement and self-expression, as well as making the start of labor more similar to spontaneous onset. Some women were relieved to be randomized for inpatient induction, because of easy access to health providers, fetal monitoring, and for not risking giving birth before arrival.

Recently, WHO presented a new guideline stating that childbirth should not just be about keeping the mother and the child alive, but also about creating a positive birth experience^15^. Women do not separate labor induction from childbirth, it is part of the same experience, and that is why women’s experience of outpatient labor induction needs to be highlighted and assessed. Women in our study were positive about the outpatient induction process. However, they pointed out different aspects of the organization, which we have called the framework in this study, for it to work well.

The issues women found important in our study are also reported in other studies on outpatient labor induction and are present in a positive birth experience, such as receiving information in advance that is detailed and available both verbally and written^3,7,16^. However, the decision of being induced is often not made before a woman comes to the hospital, resulting in insufficient time to digest the information being given during the consultation^3^. Being given enough time to consider the information and being able to make an informed decision based on their own personal context regarding inpatient or outpatient induction of labor, are essential for women having a positive experience^3^. Sharing the information with the partner is also important, as being induced at home is a joint effort.

Similar to a study by Borreli et al.^17^, women in our study expressed the importance of having clearly written instructions available at home about the induction method, as it helped clarify uncertainties^17^ and enhanced women’s feelings of safety when at home^10^. However, written information alone was not always sufficient for the women in our study, reporting that a well-functioning support system is required for making outpatient induction a good experience. Similar to other studies, we found midwives’ accessibility and support over the phone 24/7, competence, and warm and respectful response when contacted, of great importance for diminishing women’s feelings of uncertainty for some time and giving the women the confidence to stay at home^18-20^. It was also pivotal for the women in our study to know that they were always welcome at the hospital. Similar to a study by O’Brien et al.^21^, women in our study expressed a desire to receive a phone call from a midwife to feel less anxious, more followed-up and more satisfied with the outpatient experience.

The women in our study appreciated the induction method of oral misoprostol. They described it as easy to self-administer and gentle. Similar to a study by Reid et al.^11^, women experienced having more freedom and more control over the induction process as they administrated the tablets themselves. However, most women in our study experienced having uncertainties about continuing taking the next set of tablets when having some contractions. Uncertainties about the induction method in an outpatient setting is also described by others^3,7,11^, but women in our study expressed that they were comfortable with calling the hospital for advice.

Women in our study were positive about outpatient induction, but emphasized the importance of having a choice, rather than a ‘one-size-fits-all’ approach, as well as having the freedom to change their mind during the induction process. Being given a choice enhances women’s wellbeing and improves women’s experience of induction of labor^10^. However, due to changes in hospital organization and resource use, outpatient labor induction could become standard practice in the future without women being offered the choice of inpatient labor induction^9^.

As in the study by O’Brien et al.^21^, women in our study were disappointed that they had not spontaneously gone into labor but had to be induced. However, being at home was described by the women as the next best thing to a spontaneous onset, because of the constant support from the partner, the familiarity of the home environment, the higher level of privacy, the freedom of movement and selfexpression, and their ability to carry on with their usual routines at home^3,7,11,13,21^. Despite that outpatient induction of labor gave women in our study a positive birth experience, women also described some aspects with the outpatient experience as less positive. Women worried about the wellbeing of their unborn child, that they did not have immediate access to professional support when being at home, and the fear of giving birth before arrival. Surprisingly, it seems like women being induced at home have very similar worries and questions as women in spontaneous labor^19^. Although fetal monitoring and easy access to medical expertise at the hospital enhanced women’s feelings of safety^3,7,10,21^, there are some disadvantages. As in the study by Coates et al.^3^, several women in our study found fetal monitoring a disturbance in terms of limiting their freedom of movement, that it could be time consuming as well as feeling unnecessary when everything had been normal so far. In addition, several women in the outpatient group did not miss being monitored when being at home. Instead, fetal movement was interpreted as a sign of fetal wellbeing. A suggestion might be to strengthen women to trust their own body, their own capabilities, and their own instincts about the normal processes of labor and childbirth. Outpatient induction of labor with remote continuous monitoring has been tested^21^. Women in the study by O’Brien et al.^21^, were impressed by this, but some reported that the technology alone was not sufficient, direct communication with a midwife was essential.

As in other studies, knowing that the hospital could provide medical expertise in case of emergency, in addition to having the overall responsibility, was highly valued by the women^3,10^. However, our study and previous research show that women often feel forgotten and alone in the hospital because of busy staff and a busy ward^7,21^.

### Strengths and limitations

This is a small study with only 12 participants. However, all but 2 of the women who had an outpatient induction in the pilot study were included. The contribution to the data from women with an inpatient induction was limited as they had not experienced it. The interviews were performed approximately a year after childbirth. Thus, recall bias could have affected the validity of the findings as recollection after such a long time can be inaccurate or incomplete. Still, it is considered a small risk that the women have forgotten important details from the induction process that may affect the data material. This is supported by previous studies which state that women are likely to remember their birth experience in detail, both five and ten years later^22,23^. However, recall may be differential. Especially, negative experiences and incidents seem to intensify and increase over time^22^. This could have been the case for the woman induced in hospital who had a cesarean section birth and post-partum hemorrhage.

Two authors were present at nine out of twelve interviews and the transcription was done jointly providing the opportunity to critically reflect on the interview technique and to make improvements in subsequent interviews. Having three authors working together with the analysis ensured that no meaning units were left out and allowed discussion on similarities and differences between categories, thus increasing credibility as researchers’ interpretation may vary^12^.

Most of the women were positive about outpatient induction, even before they were randomized to stay at home. In addition, some women wanted outpatient induction because of the strict restrictions to have a partner present in hospitals due to the COVID-19 pandemic. Altogether, this represents a selection bias in the inclusion process. Possibly providing a more positive view of outpatient labor induction than under ordinary circumstances. Given that this is a qualitative study, our findings cannot be generalized, as the findings do not necessarily reflect the views of all pregnant women^24^. However, the women in our study had been randomized and the views from both groups were included in our study. Furthermore, a small sample size does not mean that our findings are irrelevant. Several women gave rich descriptions of their experiences, which are valuable for further research about induction of labor in an outpatient setting.

## CONCLUSIONS

Outpatient induction of labor with oral misoprostol contributed to a positive birth experience. To ensure this, the women need available written and oral information, available direct contact number to a midwife in the hospital, and the freedom to change their minds. Outpatient labor induction should be offered to women with low-risk pregnancies as this gives them the opportunity of shared decision-making.

## Data Availability

The data supporting this research are available from the authors on reasonable request.
